# From mesenchymal niches to engineered *in vitro* model systems: Exploring and exploiting biomechanical regulation of vertebrate hedgehog signalling

**DOI:** 10.1016/j.mtbio.2022.100502

**Published:** 2022-11-22

**Authors:** Fatmah I. Ghuloum, Colin A. Johnson, Natalia A. Riobo-Del Galdo, Mahetab H. Amer

**Affiliations:** a School of Molecular and Cellular Biology, Faculty of Biological Sciences, University of Leeds, Leeds, United Kingdom; b Department of Biological Sciences, Faculty of Science, Kuwait University, Kuwait City, Kuwait; c Leeds Institute of Medical Research, Faculty of Medicine and Health, University of Leeds, Leeds, UK; d Astbury Centre for Structural Molecular Biology, University of Leeds, UK

**Keywords:** Extracellular matrix, Mesenchymal stromal cells, Hedgehog signalling, Osteogenesis, Mechanotransduction, Mechanobiology

## Abstract

Tissue patterning is the result of complex interactions between transcriptional programs and various mechanical cues that modulate cell behaviour and drive morphogenesis. Vertebrate Hedgehog signalling plays key roles in embryogenesis and adult tissue homeostasis, and is central to skeletal development and the osteogenic differentiation of mesenchymal stem cells. The expression of several components of the Hedgehog signalling pathway have been reported to be mechanically regulated in mesodermal tissue patterning and osteogenic differentiation in response to external stimulation. Since a number of bone developmental defects and skeletal diseases, such as osteoporosis, are directly linked to aberrant Hedgehog signalling, a better knowledge of the regulation of Hedgehog signalling in the mechanosensitive bone marrow-residing mesenchymal stromal cells will present novel avenues for modelling these diseases and uncover novel opportunities for extracellular matrix-targeted therapies. In this review, we present a brief overview of the key molecular players involved in Hedgehog signalling and the basic concepts of mechanobiology, with a focus on bone development and regeneration. We also highlight the correlation between the activation of the Hedgehog signalling pathway in response to mechanical cues and osteogenesis in bone marrow-derived mesenchymal stromal cells. Finally, we propose different tissue engineering strategies to apply the expanding knowledge of 3D material-cell interactions in the modulation of Hedgehog signalling *in vitro* for fundamental and translational research applications.

## Graphical abstract

**Figure:**
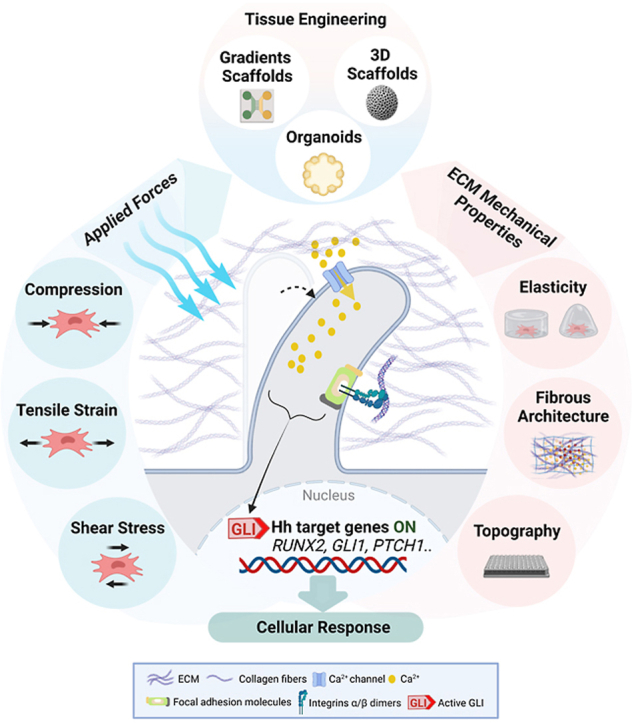

Bone formation and regeneration are complex processes that are regulated by a variety of signalling pathways, including the bone morphogenetic protein (BMP) [1], Wnt [2] and Hedgehog (Hh) pathways [3]. Hh signalling is a key regulator of bone development, MSCs differentiation and embryonic tissue patterning [3]. In this review, we discuss the crucial roles of Sonic Hh (Shh) and Indian Hh (Ihh) signalling in osteogenesis and bone development. We highlight the correlation between the activation of the Hedgehog signalling pathway in response to mechanical cues and osteogenesis in bone marrow-derived mesenchymal stromal cells (BM-MSCs). Lastly, we propose tissue engineering strategies to apply our expanding knowledge of 3D material-cell interactions in the modulation of Hedgehog signalling *in vitro* for fundamental and translational research applications.

## 1 Osteogenesis of mesenchymal stem cells

Mesenchymal stromal cells, also known as mesenchymal stem cells or multipotent stromal cells, are a subset of heterogeneous non-hematopoietic cells that can be isolated from different adult tissues such as adipose tissue, peripheral blood, and bone marrow [4]. The process of bone regeneration driven by BM-MSCs is regulated by complex interactions between various signalling pathways and the surrounding extracellular matrix (ECM) [5]. BM-MSCs can also secrete various molecular mediators with anti-apoptotic, immunomodulatory, angiogenic, and chemoattractant properties that promote bone repair [6,7]. This complex network creates the stem cell's local microenvironment or the ‘niche’ where it resides and receives stimuli from its surroundings [8], with specific architectural cues that interplay with mechanical cues to control cell fate and behaviour [9].

**Fig. 1:**
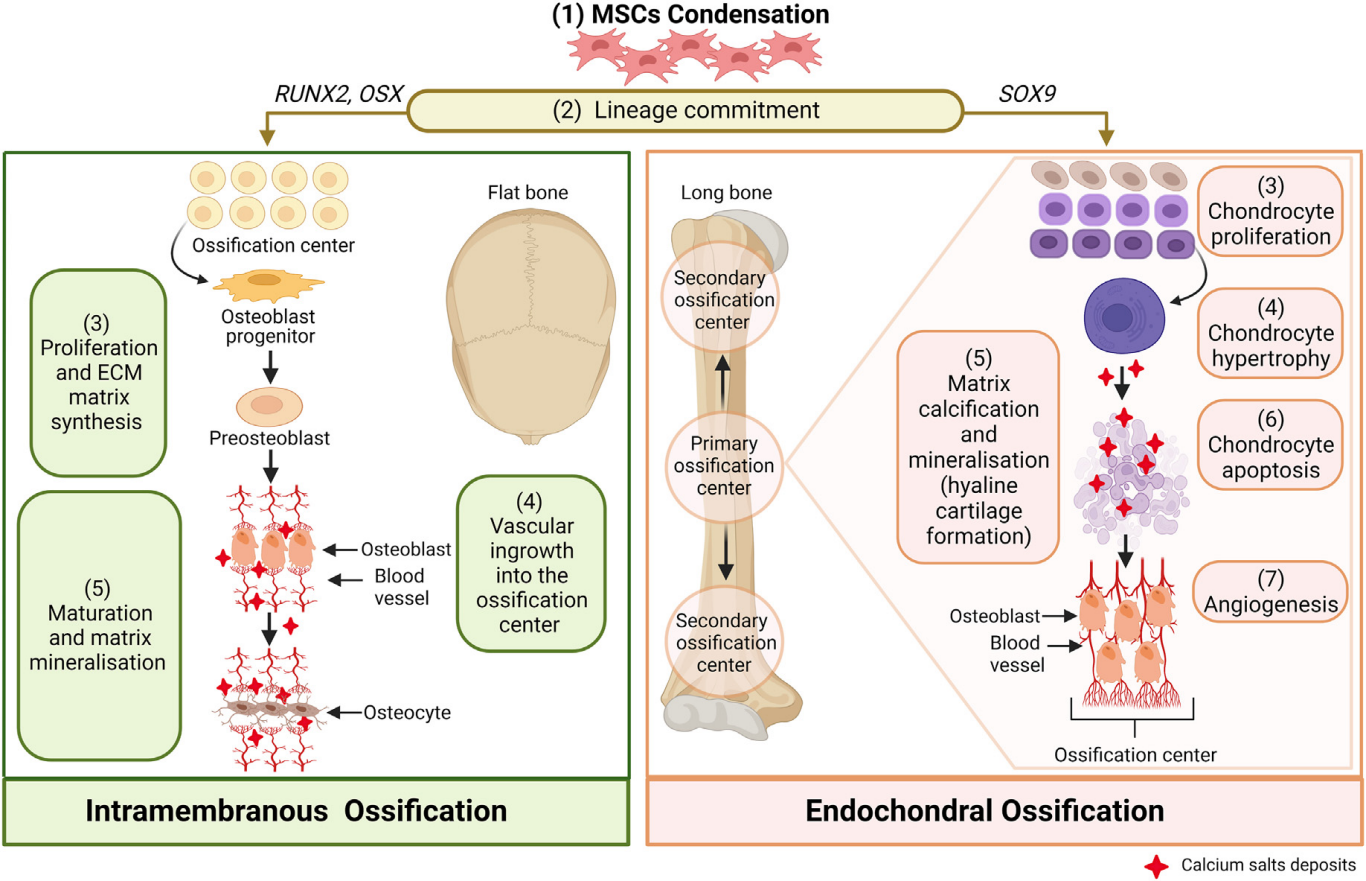
Schematic representation detailing steps involved in intramembranous and endochondral ossification processes. Both processes are initiated by the condensation of mesenchymal progenitors. In intramembranous ossification, MSCs differentiate directly into osteoblasts and deposit bone without preceding cartilage formation. A subset of osteoblasts become osteocytes upon being embedded within the bone matrix. This process occurs mainly in the flat bones of the skull and clavicle. During endochondral ossification, MSCs differentiate into chondrocytes, which then form a growth plate and undergo hypertrophy. Hypertrophic chondrocytes mineralise their matrix (mineral deposits indicated in red) and undergo apoptosis, triggering the differentiation of perichondrial cells into osteoblasts. This process forms long bones. Abbreviations: MSCs, mesenchymal stem cells; RUNX2, runt-related transcription factor 2; OSX, Osterix; SOX9, SRY-box transcription factor 9 [Created with BioRender.com].

Osteogenesis, also known as ossification, refers to the process of bone formation and development [10]. There are two principal forms of ossification: endochondral and intramembranous (Fig. 1). Both modes entail the formation of bone from existing mesenchymal tissue.

Endochondral ossification refers to the process in which cartilage intermediates are generated from BM-MSCs and then substituted by bone cells [10]. In this process, bone systematically replaces hyaline cartilage with bone tissue, forming the growing skeleton [11]. Chondrogenesis occurs as an outcome of the condensation of MSCs, followed by their differentiation into chondrocytes and secretion of typical cartilage ECM components [10]. Endochondral bone formation involves apoptosis of hypertrophic chondrocytes followed by vascular invasion from the underlying marrow, initially at the centre of the diaphysis, bringing in osteoblast precursors to form bone, thereby establishing the primary and secondary centres of ossification. These centres of ossification replace cartilage completely by the time skeletal maturity is achieved, with the exception of articular surfaces [12] (Fig. 1). There is also growing evidence for chondrocyte-to-osteoblast transdifferentiation in the endochondral ossification process [13].

By contrast, intramembranous ossification refers to the direct transformation of BM-MSCs into bone. This does not necessitate the formation of a chondrocyte template [13]. Bone formation and homeostasis is regulated by several transcription factors including SRY box transcription factor 9 (SOX9), Runt-related transcription factor 2 (RUNX2) and Osterix (OSX), which are active in chondrocytes and osteoblasts [14]. One of the best-established master transcription factors that is crucial for regulating MSCs osteogenic differentiation during intramembranous ossification is RUNX2, also known as core-binding factor subunit alpha-α (CBFA1). It is required for the osteogenic lineage commitment of the condensed BM-MSCs to osteoblast progenitors (Fig. 1) and their subsequent differentiation, maturation, and matrix mineralisation [15]. *RUNX2* is expressed in undifferentiated MSCs, and the expression is regulated by two promoters, P1 and P2, leading to two isoforms differing in the amino-terminal sequences: type I and type II RUNX2 [16]. The latter is highly expressed in all osteoblast lineage cells and thus it is considered an osteoblast-specific transcription regulator [17]. The role of RUNX2 in regulating downstream genes that determine osteoblast phenotype and controlling the expression of osteoblastogenic markers has been extensively investigated [17,18].

RUNX2 can induce commitment of MSCs into osteoblast lineage cells mainly through the activation of the Hh signalling pathway by directly regulating the expression of its key mediators: IHH, Patched1 (PTCH1) and glioma-associated oncogene homolog 1 (GLI1) [17]. GLI1 can synergise with RUNX2 by up-regulating *RUNX2* expression and further enhances its osteoblastogenic activity in MSCs and osteoblasts [19,20].

The osteogenic commitment of MSCs is regulated by biochemical and mechanical cues within their niche, which then activates various signal transduction cascades. These signalling pathways, which include Hh, Wnt, BMPs, Notch, neural epidermal growth factor-like 1 protein (NELL-1), and mitogen-activated protein kinases (MAPKs), regulate the activation of osteogenic transcription factors and nuclear mediators [21]. Furthermore, biophysical cues provided by the extracellular matrix and mechanical forces arising from physical activity are important determinants of bone growth [22]. Mature osteoblasts produce large amounts of extracellular proteins due to their strong basophilic cytoplasm, abundant mitochondria, and large Golgi apparatus [23]. The terminally-differentiated osteocytes, which are osteoblasts that have become entrapped in the bone matrix, are major regulators of bone remodelling by sensing and responding to physical signals, such as mechanical loading [24,25].

## 2 Hedgehog signalling in osteoblast-lineage cells

### 2.1 Major components of the Hedgehog signalling pathway

**Fig. 2:**
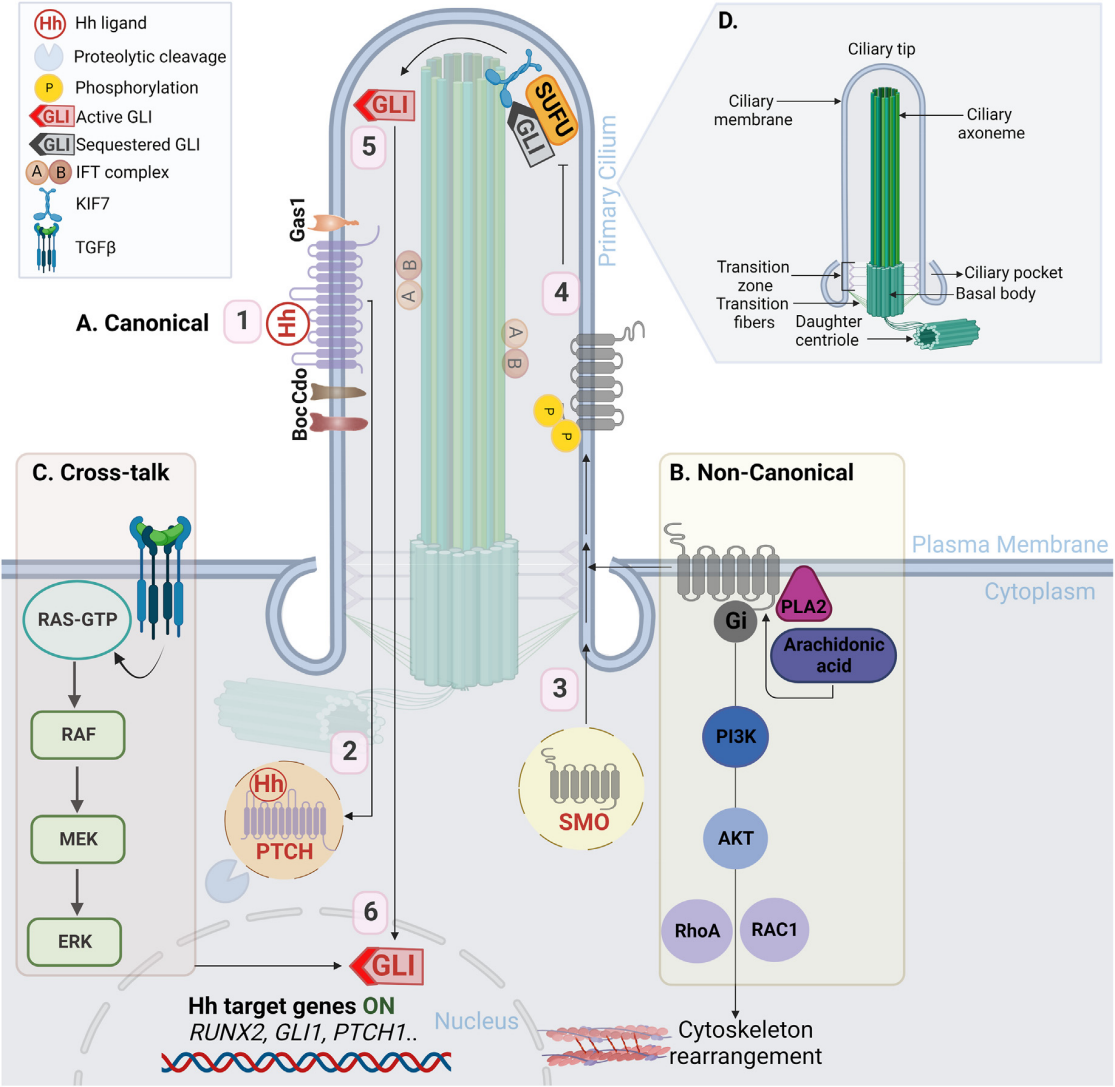
Overview of the Hh signalling pathway in vertebrates. **A)** Activated canonical Hh signalling pathway involves [1] Hh ligands binds to PTCH1 [2] leading to its internalisation and proteolytic degradation, which activates SMO [3]. The phosphorylation of SMO causes a conformational change that promotes its stabilisation and translocation to the primary cilium, where it stimulates Gi protein activity [4]. SMO activity in the primary cilium allows the release of sequestered GLI (GLI2/3) from an inhibitory complex with SUFU (5; mediated by KIF7) and [5] the active full length transcriptional activator GLI2/3 relocate to the nucleus to [6] regulate the expression of Hh target genes, including GLI1, PTCH1, and RUNX2 [7]. **B)** Examples of the SMO-dependent non-canonical activation of Hh signalling pathway. **C)** Activation of GLI1 via cross-talk with other pathways, e.g. TGFβ and MAPK/ERK signalling pathways. **D)** The structure of the primary cilium. Abbreviations: Hh, Hedgehog; P, phosphorylation; SMO, Smoothened; PTCH1, Patched1; Cdon/Cdo, cell adhesion molecule-related/down-regulated by oncogenes; Gas1, growth arrest-specific gene1; Boc, brother of Cdon; SUFU, suppressor of fused; KIF7, kinesin family member7 motor protein; IFT complex, intraflagellar transport A and B; RUNX2, runt-related transcription factor 2; GLI1, glioma-associated oncogene homolog 1; MEK, mitogen-activated protein kinase; ERK, extracellular signal-regulated protein kinases; TGFβ, transforming growth factor beta; PI3K, phosphoinositide 3-kinase; PLA2, cytosolic phospholipase A subclass 2. [Created with BioRender.com].

Hedgehog proteins regulate tissue patterning during embryonic development [26,27], and provide support for stem cell maintenance and tissue regeneration [28,29]. Vertebrates mainly express three Hh-related proteins, with mammals expressing three Hh homologues encoded by different genes: Indian (IHH), Sonic (SHH) and Desert Hedgehog (DHH). Although the core components and general mechanisms of the Hh signalling pathway are highly conserved among different species, it is more complex in vertebrates than in invertebrates, requiring additional components and organelles [30] (Fig. 2). However, the Hh pathway can be simplified into four main components (detailed in Table 1): a) Hh ligands; b) Patched (Ptch) twelve-pass transmembrane proteins; c) a seven-pass transmembrane (7-TM), G protein-coupled receptor, Smoothened (Smo); and d) effector transcription factors, the Gli family of proteins [31].

**Table 1:** A list of Hh signalling components in vertebrates with their main function(s) in the pathway.

Role	Functions	References
**Ligands**		

Sonic hedgehog (SHH)	•Expressed in many embryonic and regenerating tissues. •Plays critical roles in cell growth, normal patterning of limbs and development of bone and central nervous system.	[32]
Indian hedgehog (IHH)	•Expressed in pre- and hypertrophic chondrocytes. •Plays a key role in driving the formation of osteoblasts from hypertrophic chondrocytes during endochondral ossification.	[33]
Desert hedgehog (DHH)	Expressed in male testis, skin, and controls the formation of the peripheral nerve sheath.	[34]

**Receptors**		

Patched1 (PTCH1)	Interacts and binds to the Hh proteins to initiate pathway activation.	[35]
Patched2 (PTCH2)	•Poorly characterised and dispensable in embryogenesis. •Conflicting data regarding its activities. •Inhibits Hh signalling and response to Hh ligands.	[36]

**Co-receptors**		
Cell-adhesion-molecule related/downregulated by oncogenes (Cdon/Cdo)	Promote binding of Hh proteins to PTCH1 and can participate in negative feedback loops regulating Hh activity.	[37]
Growth arrest-specific gene1 (Gas1)		
Bother of Cdon (Boc)		

**Other Positive Regulators**		

Hedgehog acyltransferase (HHAT)	Hh palmitoylation	[38]
Dispatched (Disp)	Hh release	[39]
Protein phosphatase4 regulatory subunit2 (Ppp4r2)	Promotes dephosphorylation of SUFU, leading to its degradation.	[40]

**Other Negative Regulators**		
Protein kinase A (PKA)	•GLI phosphorylation •Promote proteolytic processing of GLI3 and GLI2 forming repressor forms.	[41,42,43]
Casein kinase1 (Ck1)		
Glycogen synthase kinase3 beta (GSK3β)		
Hedgehog-interacting protein (Hhip)	Sequesters Hh ligands	[44]
Ras-associated binding protein (Rab23)	Obstruct GLI-SUFU ciliary trafficking	[45]
Suppressor of fused (SUFU)	GLI cytoplasmic sequestration (GLI-SUFU complex)	[46]

**Signal transducer**		
Smoothened (SMO)	•Signal transducer	[47,48]
	•Member of the GPCR superfamily	
	•Sequesters PKA catalytic subunits	
**Signalling Intermediates**		

Kinesin family member7 motor protein (KIF7)	Proteolytic processing, translocation regulator and mediates the dissociation between GLI and SUFU upon Hh activation.	[49]
Kinesin Family Member3A (KIF3A)	Primary cilia assembly and biogenesis	[50]
Intraflagellar transport88 (IFT88)	GLI-SUFU complex translocation to the primary cilium upon Hh activation.	[51]
Intraflagellar transport172 (IFT172)		[52]

**Transcription factors**		
Glioma-associated oncogene homolog1 (GLI1)	Strong transcriptional activator (GLIA)	[53]
Glioma-associated oncogene homolog2 (GLI2)	Can act as a transcriptional activator or repressor (GLIA/GLIR)	
Glioma-associated oncogene homolog3 (GLI3)	Strong transcriptional repressor (GLIR)	

Abbreviations: SHH, Sonic Hedgehog; IHH, Indian Hedgehog; DHH, Desert Hedgehog; PTCH1, Patched1; PTCH2, Patched2; Cdon/Cdo, cell-adhesion-molecule related/downregulated by oncogenes; Gas1, growth arrest-specific gene1; Boc, brother of Cdon; HHAT, Hedgehog acyltransferase; Disp, dispatched; Ppp4r2, protein phosphatase4 regulatory subunit2; PKA, protein kinase A; Ck1, casein kinase1; GSK3β, glycogen synthase kinase3 beta; Hhip, Hedgehog-interacting protein; Rab23, ras-associated binding protein; SMO, Smoothened; KIF7, kinesin family member7 motor protein; KIF3A, kinesin family member3A; SUFU, suppressor of fused; IFT88, intraflagellar transport88; IFT172, intraflagellar transport172; GLI1, glioma-associated oncogene homolog1; GLI2, glioma-associated oncogene homolog2; GLI3, glioma-associated oncogene homolog3; GLIA, GLI activator; GliR, GLI repressor; PDGF, platelet-derived growth factor; GPCR, G-protein-coupled receptors.

Gli proteins act as essential effectors of the Hh signalling cascade. The Gli family regulate specific target genes involved in vertebrate developmental processes, including MSCs proliferation, differentiation, and survival [54]. Osteoblasts in primary and secondary ossification centres (Fig. 1) found in murine models are largely descendants off Gli1-expressing (Gli1^+^) metaphyseal mesenchymal progenitors and embryonic Gli1^+^ cells, respectively [55]. Gli1 is considered as a molecular marker of osteogenic lineage commitment among the majority of mesenchymal progenitor cells [55]. In the bone marrow, Gli1^+^ cells are present in the endosteal and perivascular niches, which are associated with different mechanical properties, and suggests that Gli1^+^cells may relate to distinct cell populations [56]. It has been reported that osteoblasts in secondary ossification centres are generally descendants of embryonic Gli1^+^ cells, while Gli1^+^ ‘metaphyseal mesenchymal progenitors’ located directly below the growth plate produce cancellous bone osteoblasts in the primary ossification centre [55]. Teriparatide, an osteoporosis medication, promotes the proliferation and osteoblast differentiation of Gli1^+^ progenitors. Additionally, active Hh signalling in Gli1^+^ progenitors was reported to mediate the anabolic effect of teriparatide [57]. It has also been shown that intra-articular injection of Gli1^+^ meniscal cells or purmorphamine (a Smo agonist) following meniscal injury in murine models, enhanced bone repair by promoting proliferation and migration of meniscus mesenchymal progenitors [58].

### 2.2 Canonical and non-canonical Hedgehog signalling

There are two routes of activation of the Gli transcription factor family: a) Hh ligand and Smo-dependent, which defines the classical canonical route in normal cells and ciliated tumours; and b) Smo-independent route via cross-talk with other signalling pathways, such as Transforming Growth Factor beta (TGFβ), MAPK and phosphoinositide 3-kinase (PI3K)/Akt signalling [59]. All Hh-dependent tumours have been found to display increased *Gli1* expression [60]. Moreover, both routes and multiple mechanisms of non-canonical activation may co-exist in some cancer subtypes [59].

#### 2.2.1 Canonical Hh signalling

Canonical Hh regulation (Fig. 2) has been reviewed extensively [61,62]. Briefly, canonical Hh signalling is initiated by the binding of a Hh ligand to the receptor complex containing Ptch1 or Ptch2 and at least one other co-receptor: cell-adhesion-molecule related/downregulated by oncogenes (Cdon/Cdo), brother of Cdon (Boc), or growth arrest-specific gene1 (Gas1) in the primary cilia membrane, which induces Ptch1 internalisation and degradation. Removal of Ptch1's inhibitory cholesterol mobilisation activity allows Smo activation and ciliary accumulation. The mechanism of Smo activation is controversial and appears to present species-specific differences, but it involves phosphorylation of its C-tail by G protein-coupled receptor kinase 2 (GRK2) and other kinases, and the recruitment of β-arrestin [63]. Smo translocation to the primary cilium is mediated by members of intraflagellar transport (IFT) complexes A and B, and kinesin family (KIF) motor proteins, forming a Smo-β-arrestin complex at the tip of the primary cilia [64,65]. Activated Smo stimulates heterotrimeric Gi family proteins and reduces cyclic adenosine monophosphate (cAMP) levels through its 7-TM core, promoting the release of Gli2 and Gli3 from a complex with suppressor of fused (Sufu) at the ciliary tip [66,67]. Subsequently, the full length Gli2 and Gli3 proteins, which act as transcription activators, translocate to the nucleus to regulate the transcription of Hh target genes, including induction of Gli1 expression as an auto-regulatory positive feedback loop mechanism that further propagates Hh activation [68,69]. Gli1 expression, therefore, serves as a marker for Hh signalling activity. Gli1 transcriptional activity also upregulates the expression of the signalling repressor, Ptch1, which acts as a negative feedback loop embedded within the pathway [70]. Positive and negative feedback loops are directly regulated by Gli1-dependent transcription as the terminal effect of the Hh signalling cascade [41]. Abnormal activation of Gli1 in terminally-differentiated cells is a known oncogenic biomarker in multiple cancer subtypes such as leukaemia, basal cell carcinoma, medulloblastoma and osteosarcoma making it an ideal drug discovery target [71,72]. In the absence of Hh ligands, the constitutively-expressed Gli2 and Gli3 transcription factors are converted into shorter N-terminal transcriptional repressors upon phosphorylation by protein kinase A (PKA), glycogen synthase kinase 3 beta (GSK3β) and casein kinase 1(CK1), and partial proteasomal processing. The truncated proteins retain the DNA-binding domain and repress the transcription of Hh target genes [73].

#### 2.2.2 Non-canonical Hh signalling

A balance between canonical and non-canonical Hh signalling activation is crucial in bone development to maintain homeostasis. Here we define non-canonical Hh signalling as Hh pathway effects that are independent of activation of the Gli transcription factors but are elicited by Ptch1 or Smo. Cohort studies have revealed evidence that the two modes of Hh activation can be interconnected and spatially overlap [[74], [75], [76], [77], [78]]. For example, Smo-dependent signalling stimulates the production of arachidonic acid by cytosolic phospholipase A subclass 2, which in turn binds to the extracellular cysteine-rich domain of Smo and stimulates ciliary trafficking, hence activating the canonical route of Hh signalling [74]. Smo/Gi protein signalling also stimulates the small GTPase RhoA [79]. In this context, IFT80 is necessary for osteoblastic differentiation by balancing the canonical (Smo/Gli) and non-canonical (Smo/Gi–RhoA) pathways [80].

#### 2.2.3 Cross-talk between pathways

While Ptch1, Smo, Gli and associated factors play key roles in Hh signalling, other molecular mechanisms that bypass this canonical pathway have been investigated. For example, the activation of the Gli transcription factor family can occur through cross-talk with other signalling pathways in the absence of Hh ligands and independently of primary cilia [81]. This route is regulated by several signalling pathways such as MAPK, specifically through RAS, the mitogen-activated protein kinase (MEK1/2) and extracellular signal-regulated protein kinases 1 and 2 (ERK1/2) [82]. Additionally, TGF-β and Wnt signalling pathways show cross-talk with Hh signalling [83,84].

### 2.3 Roles of IHH and SHH in bone development and regeneration

Sonic hedgehog (Shh), Indian hedgehog (Ihh), and Desert hedgehog (Dhh) are important for tissue patterning [85]. Shh is mainly involved in limb patterning, while Ihh plays a key role in skeletal development, particularly in endochondral ossification [86].

Ihh and Shh have been implicated to all aspects of bone development and regeneration, including formation and resorption. In tissue regeneration, the activation of Hh signalling can enhance osteogenesis from undifferentiated BM-MSCs, while its inhibition can promote the formation of articular chondrocytes [87]. All Hh ligands bind to Ptch1 with similar affinities but exhibit different potencies for stimulating osteoblasts differentiation in embryonic mice, with the highest potency recorded for Shh, followed by Ihh then Dhh [88].

Treatment with cyclopamine (a Smo inverse agonist) was reported to significantly decrease the expression of osteogenesis-related markers, including alkaline phosphatase (ALP), osteocalcin (OCN), osteopontin (OPN), and the BMP-9-induced transcriptional activity of Smad1/5/8, whereas the expression levels of these molecules were remarkably up-regulated by purmorphamine, which was reported to be a direct Smo agonist [89].

Shh is one of the key morphogens involved in the developmental pattern formation of various organs, such as the nervous system and limbs [90]. Previous studies conducted on chicks and mouse embryos demonstrated the importance of Shh signalling for proper limb bud patterning [91,92]. High *Shh* expression in the posterior margin of the wing bud of chicks was reported in early embryonic development, specifically in the zone of polarising activity (ZPA), which is one of the major signalling regions for MSCs differentiation [93]. Zeng *et al.* reported that Shh signalling can affect the limb bud antero-posterior patterning in both concentration and time-dependent manners, which was concluded by measuring the concentration gradient of *Gli3* expression in slices taken from different positions across the bud [94]. While some studies report the synergistic effect of BMP and Shh signalling on osteogenic differentiation [95], others have reported that the activation of Shh signalling by exogenous recombinant human Shh-N protein inhibited the osteogenic differentiation of stem cells from apical papilla [96].

Ihh is well-known for its role in endochondral ossification. During normal limb development, Ihh begins to be expressed in the condensing mesenchyme of digits when Shh signalling weakens after digit patterning [97]. However, in *Shh-*mutant mice, *Ihh* was found to be expressed at the tip of these *Shh*-mutant digits, possibly providing redundancy for *Shh* and enabling the formation of distal structures [98]. Mice deficient in Ihh display a complete lack of osteoblasts within their endochondral skeletons, although intramembranous osteoblasts form. *Ihh-*null mice were also observed to have smaller skull bones with reduced mineralisation and impaired growth of long bones with severely shortened limbs [99]. When *Ihh* was downregulated in murine osteoblasts, it resulted in decreased ALP activity and mineral deposition, as well as cell cycle arrest, which markedly increased the rate of apoptosis [100]. Ihh treatment has been reported to induce osteogenesis in mesenchymal cells by spatiotemporal regulation of Runx2 expression through Gli2 [19]. Ihh is also expressed by pre- and early hypertrophic chondrocytes to promote osteoblast differentiation [99]. It directly regulates osteoblast specification and chondrocyte proliferation, while it indirectly regulates chondrocyte hypertrophy via a negative feedback loop with parathyroid hormone-related protein (PrTHrP) [33]. The absence of Ihh in mesenchyme cells promotes the ossification of the intermediate cartilage scaffold and prevents formation of the growth plate, causing abnormal limb development [101]. Jacob *et al*. reported that constraint-induced craniosynostosis in mice is associated with higher expression of *Ihh* but not *Shh* [93]. Similarly, higher expression of *IHH* was detected relative to normal sagittal sutures controls in human craniosynostosis patients of an average age of 4.5 months [102].

These studies indicate that both Ihh and Shh signalling are crucial for osteogenesis and calvarial ossification. In summary, *Shh* expression increases at the initial developmental stages to regulate growth and patterning, while *Ihh* expression increases during the late stages of limb development in endochondral bone formation [103].

### 2.4 Hedgehog signalling and bone disease

The dysregulation of Hh signalling can have significant impact on the ossification process and lead to serious deterioration in bone homeostasis. This results in a range of bone disorders such as progressive osseous heteroplasia [103], osteoarthritis [104], osteoporosis [105], and bone fractures [106]. In addition, over-expression of Hh pathway core components in adult tissues is linked to cancer [107]. *IHH*, *PTCH1* and *GLI1* expression levels were strongly associated with each other and with tumour proliferation in human osteosarcoma cell lines and primary tumour specimens [108].

Hh expression has also been shown to enhance osteogenesis and mineralisation in human induced pluripotent stem cell (iPSC)-based models derived from individuals affected by Gorlin syndrome and McCune-Albright syndrome (Table 2), which show opposite characteristics in terms of bone mineral density and Hh activity [109]. The findings suggested that Hh signalling is a key controller of osteoblastic differentiation from precursor cells. Table 2 presents examples of bone-related disorders and tumours caused by dysfunctional Hh components.

**Table 2:** Selected examples of bone developmental disorders and cancers caused by dysfunctional Hedgehog signalling components.

HH Component	Bone Disorder	Symptoms	OMIM	References
IHH	Brachydactyly type A1	Shortened and often malformed digits of the hands	112500	[110]
	Acrocapitofemoral Dysplasia	Cone-shaped epiphyses in hands and hips, relatively large head and narrow thorax	607778	[111]
SHH	Holoprosencephaly 3	Abnormal brain and facial structure; midfacial clefts such as cleft lip and palate, cyclopia (single eye)	142945	[112]
	Schizencephaly	Developmental delay, ​seizures, problems with brain-spinal cord communication, partial or complete paralysis, poor muscle tone	269160	[113]
	Postaxial polydactyly type A1 and B	Congenital hand and foot deformities	174200	[114]
GLI2	Culler-Jones syndrome	Hypopituitarism and/or postaxial polydactyly	615849	[115]
	Holoprosencephaly 3	Abnormal brain and facial structure; midfacial clefts such as cleft lip and palate, cyclopia (single eye)	142945	[112]
GLI1	Postaxial polydactyly type A8	Presence of postaxial extra digits (hexadactyly) on the hands and/or feet	618123	[116]
	Preaxial polydactylyI	Polydactyly on the radial side of the hand	174400	[117]
PTCH1	Gorlin's syndrome	Cortical defects of bones with large body size, bisecting ribs and wedge-shaped vertebrae, basal cell carcinomas and cerebellar medulloblastomas.	109400	[118]
	Holoprosencephaly 7	Mental retardation and craniofacial malformations	610828	[119]
	Brachydactyly type A1	Shortened and often malformed digits of the hands	112500	[110]
SMO	Curry-Jones Syndrome	Multisystem disorder: Patchy skin lesions, polysyndactyly, diverse cerebral malformations, unicoronal craniosynostosis, and intestinal malrotation with myofibromas or hamartomas	601707	[120]
SUFU	Gorlin's syndrome	See above	109400	[118]
KIF7	Al-Gazali-Bakalinova syndrome	Multiple epiphyseal dysplasia, macrocephaly and distinctive facial features: frontal bossing, short neck, spindle-shaped fingers and prominent joints.	607131	[121]
IFT172	Short-rib thoracic dysplasia-10 with or without polydactyly	Constricted thoracic cage, short ribs, shortened tubular bones, and a ‘trident’ appearance of the acetabular roof.	615630	[122]
**Cancers**				
PTCH1, PTCH2	Rhabdomyosarcoma	Soft tissue sarcoma	–	[123]
IHH, PTCH1, GLI1	Osteosarcoma	Primary bone cancer	259500	[108]

Abbreviations: OMIM, Online mendelian inheritance in man; IHH, indian hedgehog; SHH, sonic hedgehog; GLI, glioma-associated oncogene homolog; PTCH, patched; Disp, dispatched; SMO, Smoothened; SUFU, suppressor of fused; KIF7, kinesin family member 7 motor protein; IFT172 intraflagellar transport 172; Cdon/Cdo, cell-adhesion-molecule related/downregulated by oncogenes; Gas1, growth arrest-specific gene 1; Boc, brother of Cdon.

Activation of Gli1 transcription factors beyond Smo contribute to the development of several types of tumours with elevated Gli1 activity [124]. In bone malignancies such as osteosarcoma, the MAPK pathway promotes transcriptional activation of *Gli1* and post-translational modifications of Gli1, leading to elevated Gli1 oncogenic activity [59]. ERK2 MAP kinase has been reported to directly phosphorylate Gli1 on multiple target sites near the SUFU-binding motif, which weakens the binding of SUFU to GLI1 and facilitates the release of SUFU [125]. Furthermore, MEK1/2 promotes Gli1 transcriptional activity, which is lost by MEK1/2 inhibition *in vitro* [59]. Thus, several clinical trials have been established targeting MAPK signalling, especially those elicited by the MEK1/2-ERK1/2 pathway for cancer therapy [126]. Emerging data suggests that modulating Hh signal transduction may reduce osteosarcoma growth and stop osteosarcomagenesis [127].

To assess the involvement of Hh signalling pathway components in disease, Hh signalling is typically evaluated by measuring *Gli1* gene expression levels using either reverse transcriptase (or real-time) polymerase chain reaction (RT-PCR). Additional *in vitro* cell-based assays employed have included stroma-cancer (luciferased) co-cultivation assays [128], phenotypic growth assay of irradiated Ptch+/- cells [129], a cell-free membrane binding assay for Smo [130], and Gli–luciferase reporter gene assays [131]. Tissue-based assays are another *in vitro* approach that allow monitoring of the tumour progression, for example by utilising chick neural plate explants [130]. *In vivo* models can provide more accuracy in representing bone diseases and tumours caused by aberrant Hh activation [132]. Therefore, several murine models have also been developed to achieve this, including *Ihh*-null mouse models [99], mouse models heterozygous for PTCH1 [133], *Ptch*+/- medulloblastoma models [134] and human xenograft models [[135], [136], [137]].

By contrast, upregulation of Hh signalling occurs during bone healing, with Hh morphogens reported to play important roles in promoting osteogenesis and angiogenesis during bone repair. In contrast to BMP2 signalling, Hh signalling negatively regulates adipogenesis [138] and does not produce excessive ectopic bone when placed at the site of interest [139]. This makes promoting Hh signalling of interest for regenerative medicine applications as an alternative to BMP2.

## 3 Mechanical regulation of the Hedgehog signalling pathway

Cells can sense and react to changes in the physical properties of its extracellular environment [140]. Despite considerable variation in tissue architecture, stem cells in different organs interacts with their ‘niche’ to transition between regenerative and quiescent states. The differentiation of MSCs into specific lineages as a function of substrate stiffness highlights the key role of the physical environment in directing cell fate and organ formation [141].

Mechanobiology is the study of cell behaviour in response to external mechanical loadings, such as tension, compression, fluid shear stress, hydrostatic pressure, and ECM properties [142,143]. Mechanical cues have been implicated in the determination of stem cell lineage commitment, proliferation, differentiation, migration, and apoptosis [144]. The unique features of Hh signalling, including long-range diffusion and cross-talk with other signalling pathways, suggest its modulation by biophysical cues [90]. The ability of a cell to sense and respond to mechanical cues is governed by mechanosensitive receptors (mechanosensors), which adapt to the physical properties of their microenvironment and translate external mechanical cues into biochemical signalling events in a process known as mechanotransduction [50]. Some proteins respond to mechanical forces via changes in the binding affinities for associated proteins. This serves as a mechanical link for transmission of extracellular forces into the cell and *vice versa*, and does not involve the direct induction of biochemical signalling [145,146]. Other proteins provide scaffolding for phosphorylation events in response to mechanical stretching [147,148] or modulate enzymatic activity, which can trigger mechano-chemical signalling pathways [149,150]. Several macromolecular complexes (mechanotransducers) have been identified, including cytoskeletal- and nucleoskeletal-related proteins, adherens junctions, integrins, primary cilia and ECM proteins [151].

Mechanotransduction can be initiated via chemical [152] and mechanical [153] cues that regulate MSCs differentiation [154]. Mechanical cues, including substrate elasticity (stiffness), surface topography and mechanical forces such as tension, compression and fluid flow, can direct MSCs function and fate even in the absence of biochemical factors [[155], [156], [157], [158], [159], [160], [161], [162]].

The mechano-response of MSCs integrates events at the molecular, cellular, and multicellular levels, and occurs at timescales ranging from milliseconds to days [163]. At the cellular scale, mechanobiology specifies how MSCs sense, decipher and respond to mechanical loading. At the molecular level, mechanobiology determines how mechanosensors are recruited and connected together to activate downstream signalling pathways and regulate the expression of osteogenesis-related genes [164]. Differential gene expression analysis by RNA-sequencing revealed high expression levels of Wnt and Hh signalling target genes as a mechanoresponse to tibial compression in cortical bone [165]. In fact, Hh signalling inactivation has been reported to be directly related to impaired tendon development and the inability to adapt to altered mechanical loadings [166]. Furthermore, murine models with conditional *Smo* deletion in tendons have been shown to display increased yet dysfunctional primary cilia, with diminished mechanoresponsiveness to external mechanical loading [167]. Surprisingly, mechanical overload was able to stimulate Hh activation even in the absence of the primary cilia in tibial plateau chondrocytes, suggesting a mechanical loading-dependent adaptation in chondrocytes *in vivo* [168].

Irrespective of location and developmental mechanism (i.e. intramembranous or endochondral), Hh signalling is required for osteoblast differentiation during pre- and post-natal bone growth. Mechanical loading is known to regulate bone development, homeostasis, remodelling and adaptation [169]. Loss of mechanical stimulation can affect various stages of skeletal development including patterning, differentiation, growth and morphogenesis, thereby leading to bone weakening and increasing risk of bone fracture [170]. Furthermore, bones are shaped according to the mechanical force exerted by muscle contraction and gravity during embryonic development [171]. In early bone development, osteogenesis-related gene expression is regulated by a range of cytokines, growth factors, and hormones [172]. It can also be activated by mechanical stimuli such as fluid flow shear stress generated by bone loading across the surface of bone cells [173]. These early changes contribute to the induction of osteoblast differentiation, chondrocyte maturation and skeletal morphogenesis [[174], [175], [176]]. Pharmacological immobilisation of embryonic chicks has also shown that motility is a key factor that determines the rate of skeletal growth [177]. In humans, foetal movements during pregnancy generate mechanical loadings (in the form of stress and strain) which is critical for normal prenatal musculoskeletal development [178]. Restrictions to this movement may lead to multiple congenital disorders [179].

Intramembranous bones contain less bone marrow compared to endochondral bones and thus respond differently to mechanical loadings [180,181]. Muscular activity and rapid expansion of the brain during its growth generate mechanical strain that directly affect the growth and shape of a number of intramembranous bones [181] and induce ossification centres [182]. Muscle contraction has been found to regulate the shape and size of the proliferative chondrocytes during endochondral ossification which directly influenced cartilage morphology [183]. It has also been reported that mechanical stress generated by cyclic tensile strain altered the expression of ECM in human articular chondrocytes [184]. In chondrocytes, mechanically-induced cilia disassembly inhibits Hedgehog signalling [185]. Overall, a key element for normal bone development and articular cartilage function is maintaining the mechanical properties of cartilage-bone interfaces [186].

### 3.1 The primary cilium as a mechanosensor

The primary cilium is a long, thin organelle extending from the surface of almost all mammalian cells. Almost every cell in the body assembles one primary non-motile cilium during interphase [187]. Primary cilia control mechanical stimulation with a dual-sensing ability, acting as mechanosensors and chemosensors [188]. Specialised cilia of photoreceptors also act as photosensors [189]. They link the cell membrane to the ECM and the nucleus and regulate downstream signalling pathways.

Primary cilia consist of a microtubule-based axoneme of nine doublet microtubules that extends from a basal body (Fig. 2). Its unique structure is ideal for sensing external mechanical loadings by acting as cAMP-responsive mechanosensors in MSCs [190]. Furthermore, shear forces cause primary cilia to deform and bend, leading to calcium influx which regulates cell size and mediates cell-cell interactions [191]. Ciliary bending has been reported to be influenced by both length and flexural stiffness of the axoneme [192], and the degree of ciliary bending has been shown to regulate the strength of the Hh signalling pathway response [193].

Cilia is the main organelle which coordinates with the Hh signalling pathway to transmit mechanical signals [194]. Gli1 transcription is suppressed by the formation of Sufu-Gli2 and Sufu-Gli3 complexes at the tip of the primary cilia in the absence of Hh ligands [194]. Furthermore, Ptch1 and Smo transmembrane proteins are localised on the cilium membrane and they either move into (Smo) or out (Ptch1) of the cilium in response to the presence of Hh proteins [195]. These movements are facilitated by IFT complexes and IFT motor proteins kinesin family member 3A (KIF3A) which are required for ciliogenesis [196]. Mutations in various IFT proteins displayed accumulation of Gli2 and Gli3 at the cilium tip [197] and disrupted Smo localisation to the cilium, thereby blocking canonical Hh signalling but enhancing non-canonical signalling in differentiating osteoblasts by increased levels of extra-ciliary Smo [198]. While the position and the temporary presence of the primary cilium may limit the timing and spatial dynamics of ligand reception, these limitations have been suggested to offer the potential for spatiotemporal regulation of Hh signalling. For example, this localisation would make it possible for some cells to be sensitive to the ligand while others in a certain setting would not be [31].

Several studies have reported that non-canonical Hh pathway functions outside primary cilia but can occur in parallel to canonical signalling initiated by ciliary Smo [76,79,199]. Primary cilium-mediated mechanotransduction has been shown to be cAMP-dependent in MSCs. The G protein-coupled receptor 161 (Gpr161)-cAMP-PKA signalling axis in the primary cilium negatively regulates canonical Hh signalling and may determine the transcriptional outcome of Hh signalling in response to mechanical cues. Johnson *et al*. suggested that under fluid shear stress, Hh signalling is indirectly activated in MSCs via increased expression of Gpr161 in the primary cilium through a pathway involving adenylyl cyclase 6 (AC6) and cAMP signalling [158]. However, cilia-specific analysis is required to fully explore the role of the primary cilium in this mechanism.

It is well-known that cAMP fluxes in cilia are sensed by PKA, which is a potent negative regulator of Hh signalling [199]. The positive effect of PKA on Hh signalling has been reported in studies conducted on *Drosophila* and found to be mediated through effects on Smo [[200], [201], [202]]. On the other hand, Smo in vertebrates lacks the critical PKA phosphorylation sites which implies that Smo activation is PKA-independent [203]. Thus, further investigations into the molecular mechanisms underlying the potential PKA-dependent activation of Hh signalling in MSCs under mechanical stimulation is needed.

Primary cilia and its related proteins are involved in osteoblastic differentiation and mechanical stimulation–induced osteogenesis in MSCs [204]. Interstitial fluid flow through the lacuno-canalicular system is recognised to play a key role in bone function and metabolism. The pressure gradient generated from the permeability differences between the mineralised and unmineralised matrices surrounding the osteocytes creates shear stress [205]. This shear stress is sensed by the primary cilia and their deformation triggers calcium influx, leading to an AC6-dependent reduction in cAMP [206]. Stimulated osteocytes secrete the receptor activator of nuclear factor-κB ligand (RANKL), which activates osteoclasts via RANKL-RANK binding leading to bone resorption. Bone formation by osteoblasts is then triggered by factors released from the resorbed bone [207]. Primary cilia also release extracellular vesicles, known as ciliary ectosomes, that may be involved in ciliary response to fluid flow [208], with evidence suggesting that ectocytosis prevents accumulation of excess ciliary proteins [209]. Furthermore, cilia on osteoblasts and osteocytes indirectly regulate osteoclast formation by inducing RANKL expression in the bone microenvironment [210]. The activation of ciliary signalling in mesenchyme during the development of tubular organs generates mechanical forces that also activate the Hippo pathway via Yes-associated protein (YAP, also called YAP1) and its related co-transcriptional factor TAZ, which promotes cell proliferation and inhibits apoptosis [211]. It has been shown that YAP restricts the Hh-dependent differentiation of smooth muscle [212,213]. This indicates the important role of primary cilia as mechanical responders and Hh signal transducers to regulate cellular adaptation to mechanical loadings.

### 3.2 Integrins

Integrins are heterodimeric transmembrane proteins consisting of α and β subunits [214], and are well-known for their mechanosensory function [215]. Integrins are the major cellular ECM receptors that mediate cell-to-cell and cell-to-matrix interactions. The ECM ligand-integrin-cytoskeleton linkages are known as ‘clutches’ which, when activated by mechanical cues, result in MSCs differentiation and ECM remodelling [216].

The dynamic interaction between osteoblasts and ECM is mediated by collagen type I alpha-1 chain (COL1A1) and α_2_β_1_ integrins [217,218]. COL1A1/α_2_β_1_ binding activates MAPK/ERK signalling and phosphorylates RUNX2, which increases osteogenic gene expression such as *osteopontin (OPN)* under shear force [218]. Murine pre-osteoblasts displayed absence of osteogenesis when COL1A1/α_2_β_1_ binding was disturbed by blocking inhibitors [219]. Osteogenic induction via COL1A1/α_2_β_1_ binding is not limited to shear force, as it has been reported that three-dimensional (3D) micro-topographies can induce the osteogenesis of MSCs, which displayed different integrin-mediated attachment responses on different topographically-textured surfaces. Ingenuity Pathway Analysis of this data identified the ERK, Interleukin-1 and JNK pathways as key signalling pathways involved [156].

Furthermore, it has been reported that upregulation of β_1_ activity in MSCs under mechanical stimulation activates Shh signalling, leading to upregulation of OPN, OCN, osteonectin and OSX proteins [220,221]. Nano-scale topographies were reported to upregulate markers of osteogenesis in the human MG63 ​cell line (*BMP-2, RUNX2,* *ALP* and mineralisation) in a diameter-dependent pattern via integrin β_1_ by activating the Smo-dependent Hh pathway [221]. A nanotube diameter of 70 ​nm provided the optimal environment for Shh protein production and activation of Smo, resulting in the transcription of Gli target genes. Furthermore, *Ihh* and α_5_β_1_ integrin, which are co-expressed in pre-hypertrophic chondrocytes, were found to play a critical role in the maintenance of normal articular cartilage [222]. In addition, α_V_β_1_ integrins promote osteoblast recruitment to ossification centres during endochondral ossification [223]. The α_V_β_3_ integrins have also been reported to induce osteogenesis by regulating myosin II-mediated contractility and enabling cells to sense ECM mechanics [224].

The mechanosensitive proteins that connect integrins and cytoskeletal actin fibres include focal adhesion molecules, which are highly dynamic structures that mediate cell-ECM adhesion [225]. In response to mechanical stretching, focal adhesions in MSCs instigate a cascade of mechanotransductive signalling pathways such as Ras homologous (Rho) and MAPK signalling, leading to nuclear localisation of YAP/TAZ and ERK, respectively [226], to activate RUNX2 and eventually induce MSCs osteogenic differentiation [227].

### 3.3 The extracellular matrix

The variable composition of the ECM within each tissue provides unique architectural cues which stimulate mechanosensation in MSCs during development [228]. Bone ECM is composed of 40% organic and 60% inorganic components, with the majority of inorganic components being calcium phosphates in the crystalline form of hydroxyapatite (HA). Organic components of bone ECM mainly include COL1A1, elastin, fibronectin, laminin, in addition to soluble components, comprising cytokines, growth factors and proteases [229].

Rezenkov *et al*. have identified twelve levels of bone ECM organisation, starting from the basic components of HA and collagen type I fibrils at the nanoscale to more complex structures at the macroscale level [230]. The effects of ECM on MSC fate can be summarised in two main aspects. Firstly, the biochemical aspect is determined by the chemical nature and composition of the ECM, which in turn determines its biochemical characteristics such as charge and hydrophobicity. Secondly, the mechanical aspects are determined by the impact of the elasticity and topographical architecture of the ECM, including its micro- and nano-scale features [231].

ECM elasticity is controlled by fibronectin and collagen type I composition. Fibronectin polymerisation regulates the formation of collagen fibrils which increases ECM stiffness [232]. It has been confirmed that MSCs tend to display greater adipogenic differentiation potential in softer environments while those on stiffer ones have a tendency to undergo osteogenesis [141].

The exact mechanism by which ECM stiffness influences Hh signalling pathway remains unclear. However, it has been proposed that ECM stiffness induces osteogenic differentiation of MSCs by regulating Hh signalling indirectly via integrin-mediated mechanotransduction and focal adhesion kinase [162]. ECM stiffness regulates osteogenic differentiation primarily via the relationship between surface-membrane integrins, the two mechanotransducers RhoA kinase, and focal adhesion kinase, which result in the activation of downstream signalling pathways such as PI3K/Akt and ERK-MAPK signalling pathways [[233], [234], [235], [236]]. Furthermore, ECM stiffness induces TGFβ activation which drives cellular stress and ECM synthesis [237].

In addition, fluid flow shear stress generated by bone loading [238] can stimulate integrin-mediated activation of focal adhesion kinase, leading to the activation of downstream signalling pathways such as ERK, c-Jun N-terminal kinase (JNK), p38-MAPK and the PI3K/AKT pathways [218,239]. The aforementioned pathways can potentially enhance Gli1 activity and cross-talk with the Hh signalling pathway to induce osteogenic differentiation of MSCs (Fig. 2).

The complex ECM topography is composed of micro and nano-scale patterns which direct cells mechanosensation [231]. The regulation is attributed to the size similarity with cell surface receptors, such as integrins which triggers downstream signalling and manipulates cell functions [240]. Additionally, the mechanical information arising from the micro and nano-scale patterns directs the alignment of actin fibres and microtubules at the cytoskeletal level. The impact further propagates to the nucleus to induce changes in the cell morphology, adhesion, and migration [241].

Changes in the growth plate exemplify how changes in matrix composition are regulated as a function of Hh-regulated differentiation. Ectopic expression of Ihh in proliferating chondrocytes is associated with abnormal expression of type X collagen, typically expressed by hypertrophic chondrocytes [242]. Moreover, the matrix phenotype of the growth plate is disrupted upon mutation of proteins involved in intraflagellar transport, which is responsible for assembling and maintaining cilium [243]. Reduced proteoglycan production and type X collagen expression are also observed upon deletion or mutation of proteins associated with IFT particles, such as IFT80 [244]. Additionally, cartilage-specific knockout of *Kif3a* has been reported to be associated with altered type X collagen expression in the murine growth plate [245].

Planar two-dimensional (2D) cultures are only able to reproduce up to the third or fourth level of organisation proposed by Reznikov *et al.* [230] and does not provide critical biophysical cues, such as topographical patterns which occur *in vivo* [246]. Therefore, it is crucial to develop ECM-mimicking scaffolds with appropriate surface engineering *in vitro* to enable the study of the precise mechanosensing behaviour of MSCs and cellular responses to topographic features.

### 3.4 Hedgehog signalling in response to mechanical cues *in vitro*

**Fig. 3:**
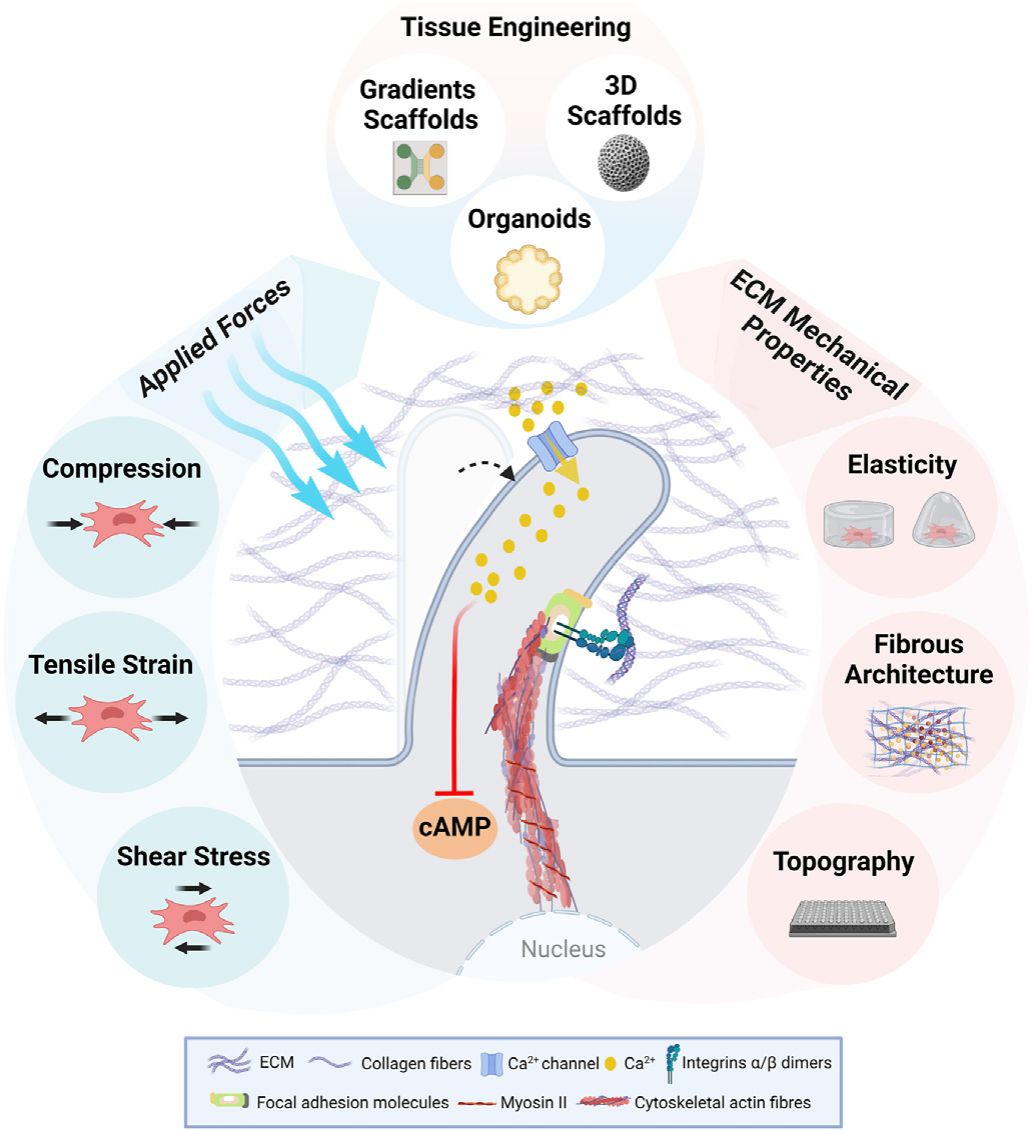
Cells are equipped with various mechanisms to sense surrounding biophysical cues and mechanical forces within its microenvironment. This can be exploited using various tissue engineering strategies, including gradient tissue-like structures, 3D scaffolds and organoids. Applied forces (in blue) can include compression, tensile strain and shear stress forces, which cause ciliary bending and Ca^2+^ influx, thereby inhibiting cAMP production. Other extracellular matrix properties (in red) include elasticity (stiffness), fibrous architecture (includes fibre orientation, ECM porosity and ligand density), and topographical cues. This ‘outside-in’ signalling is integrin-mediated: Integrin adhesion complexes bridge the extracellular matrix to the actin cytoskeleton and transduce signals in response to mechanical and chemical cues. Abbreviations: ECM, extracellular matrix; Ca^2+^, calcium ions; cAMP, cyclic adenosine monophosphate. [Created with BioRender.com].

Mechanical stimuli are sensed by cells via a range of cellular mechanosensor proteins and protein assemblies at the cell-ECM interface. Bone-residing cells are exposed to a range of extrinsic and intrinsic mechanical cues, which include fluid flow, compression, ECM stiffness and topographical features (Fig. 3). Topographical elements, such as pillars, pits and tubes, and other physical properties of the ECM have been widely studied *in vitro* by using tailored surfaces to mimic ECM features. Few studies have investigated the impact of physical cues on the Hh signalling cascade.

Table 3 presents studies that have investigated the impact of various mechanical cues on Hh signalling in different cell types, including murine embryonic fibroblasts [158,162], vascular smooth muscle cells [247], chondrocytes [161,248,249], and osteoblasts [159]. However, the underlying mechanisms regulating how these cells respond to such mechanical cues remains poorly understood. Limitations of these studies have included the lack of further insights into potential post-translational modifications [247] and lack of cilium-specific analysis of the mechanisms uncovered [158]. This presents exciting avenues for future studies in this area.

**Table 3:** Overview of Hh signalling-related studies into the role of mechanical cues in mediating cellular function and fate.

Mechanical Factor		Model	Cell Response	Reference
**Strain**	Cyclic stretch	Human ligamentum flavum cells obtained by a tissue explant method	• Increased expression of *IHH, SMO, GLI1, GLI2* and *GLI3* and markers of osteogenesis • Increased ALP activity	[157]
	Cyclic strain and pulsatile flow	Adult rat vascular smooth muscle cells	Hh expression reduced by mechanical stimulation: • Decreased proliferation and increased apoptosis. • Decreased expression of *Shh* and *Ptch1*	[247]
	Cyclic tensile strain	Primary bovine articular chondrocytes and chondrocyte cell line (from Tg737^ORPK^ mice)	• Mechanical strain upregulates Ihh expression. • Increased expression of *Gli1* and *Ptch1*	[161]
**Compression**	Hydrostatic loading	Primary rat epiphyseal chondrocytes	Increased Ihh gene expression and Ihh-responsive GLI-luciferase activity. This was obstructed by disrupting primary cilia structure.	[248]
	Static pressure	Rabbit condylar chondrocytes	Increased expression of COL2A1, SOX9, ALP, Runx2, and increased mRNA levels of *Ihh* and *PTHrP*	[249]
**Shear Stress**	Oscillatory fluid flow	Murine C3H10T1/2 ​cell line	• Increased expression of *Ptch1*, *Gli1, Runx2* and *Opn.* • Enhanced osteogenesis via a Gpr161, AC6, cAMP, Hh mechanism.	[158]
**Elasticity**	Methacrylated gelatin hydrogels	Murine embryonic fibroblasts	Varying expression of *Gli1* and β1-integrin with varying hydrogel stiffness.	[162]
**Topography**	Titanium discs with pits (ranging from 1 to 40 ​μm) and nanotubes of diameters 25 & 70 ​nm	Human MG63 (osteoblastic) cell line	Increased expression of *Smo, Gli1, Shh* on textured surfaces.	[159]

Abbreviations: RUNX2, Runt-related transcription factor 2; OSX, Osterix; ALP, alkaline phosphatase; OCN, Osteocalcin; OPN, Osteopontin; Shh, Sonic hedgehog; Ihh, indian hedgehog; SMO, Smoothened; Gli1, glioma-associated oncogene homolog 1; Gli2, glioma-associated oncogene homolog 2; Gli3, glioma-associated oncogene homolog 3; RANKL, receptor activator of nuclear factor-κB ligand; COL2A1, collagen type II alpha-1 chain; μm, micrometre; nm, nanometre.

Studies have reported that increased matrix stiffness is supportive for tumour progression [250,251]. Pharmacological interventions targeting Shh signalling (e.g., vismodegib, a Smo inhibitor) to limit ECM stiffening have been tested in clinical trials for various cancers [250,252]. Shh can activate quiescent fibroblasts to differentiate into cancer-associated fibroblasts during tumorigenesis [253], which synthesise ECM proteins [254] and ECM-crosslinking enzymes, such as lysyl oxidases [255], resulting in increased tumour ECM stiffness. Inhibiting Shh with vismodegib has been reported to improve the efficacy of chemotherapy in solid tumours such as breast and pancreatic cancers [252]. However, preclinical trials using vismodegib in patients with metastatic pancreatic cancer revealed no significant improvements with regards to drug delivery or treatment efficacy [250].

## 4 Mechanobiological design opportunities for modelling Hedgehog signalling *in vitro*

Efforts to identify Hh pathway effectors are limited by the available *in vitro* assays, which depend on the use of exogenous chemical modulators of Hh signalling, as well as the financial and ethical costs associated with animal models. To bridge the gap between basic discovery research and clinical applications, *in vitro* models need to recapitulate 3D cell-cell and cell-matrix interactions seen *in vivo* whilst also allowing for systematic experimental interventions. Cell-substrate interactions can be modulated in the laboratory to regulate cell behaviour and alter key signalling pathways, offering a step-change in our ability to study Hh signalling *in vitro*. Various strategies to achieve this are reviewed below.

### 4.1 Engineering gradient tissues and microfluidics-based systems

Tissue morphogenetic gradients are established during development and maturation [256]. These morphogen gradients in turn generate cellular and mechanical gradients by regulating cell proliferation and specification. This can be seen in osteochondral tissue for example, which is derived from gradients of morphogens such as Ihh and BMPs [257,258]. This directs the differentiation of osteo- and chondro-progenitors into a gradient of chondrocytes, hypertrophic chondrocytes, and osteoblasts [259,260]. Eventually a heterogeneous pool of ECM components secreted from these different cell populations provide the mechanical cues required for the function of osteochondral tissue [261]. A number of tissue engineering strategies have been developed that permit gradient scaffold fabrication, including a range of additive manufacturing strategies and controlled phase changes.

Controlled spatial distribution of differentiation can be achieved by stacking layers of hydrogels or 3D-bioprinting of scaffolds with gradients of pore size, electrospun fibre alignment and mineralisation [262], and/or controlled fluid deposition to provide a stepped transition gradient [[263], [264], [265], [266]]. Similarly, applied forces such as vortex mixing of biomaterials, de-mixing of fluid components, or thermally-induced convective flow can be utilised to redistribute homogenous components into controlled architectural gradients [[267], [268], [269], [270]]. Another approach to create morphogen gradients is by genetic engineering of cells to undergo self-patterning into spatially organised tissues [271] or by applying external magnetic [272], or electric fields [273] to generate mechanical gradients.

Microfluidic platforms are also being investigated to achieve linear and synchronised orthogonal or opposing gradients of developmental morphogens. Demers *et al.* generated the Shh concentration gradient seen across the neural tube by utilising a four channel-microfluidic device filled with Matrigel [274]. The dynamic spatiotemporal construct relays on the simple Fickian diffusion principle to create a linear Shh concentration gradient that induces embryonic stem cell differentiation and directed motor neuron development in a dose-dependent manner [274]. In another study, two microfluidic chips were used to assess cigarette smoke-induced malignant transformation of human bronchial epithelial cells by assessing the expression of Hh signalling proteins [275]. To achieve this, one chip included a concentration gradient-generator for the determination of the optimal concentration of cigarette smoke for cell stimulation, while the other chip was made up of four chambers with a central channel, and was used to assess the impact of long-term stimulation of cultured cells using the optimal concentration of smoke determined using the first chip. This technique can potentially be implemented for the efficient screening of target biochemical gradients.

Several studies have considered the importance of engineering tissue gradients to study the spatiotemporal function of Hh signalling in tissue patterning during embryonic development. Li *et al.* reconstituted the architectural gradient of Shh signalling *in vitro* using mammalian cells to create synthetic molecular signal gradients that mimic gradient distributions *in vivo* [271]. Similarly, a microphysiological construct mimicking the SHH concentration gradients endogenously expressed in the epithelium was developed [276]. A microplate-based platform that supports SHH ligand-secreting epithelium was placed over matrix-embedded, SHH-sensing mesenchymal cells, which recapitulated the architecture of developing orofacial tissues. The generated SHH concentration gradient directly affected the expression patterns of SHH-responsive genes in the adjacent mesenchyme. This stimulated a gradient of pathway activation in the mesenchymal layer which decreased as the distance from the epithelium increased, demonstrating that SHH concentration gradients spatiotemporally affected cellular activities [276].

Alternatively, mechanical gradients of varying stiffnesses can be generated though spatially-controlled crosslinking of hydrogels by graded light exposure [277], sliding photomasks [278] or a unidirectional freezing process [279]. Post-modification of pre-formed hydrogels can also be achieved by dip-coating [280], controlled immersion [281], controlled molecular diffusion [282], or laser-scanning lithography [283]. These fabrication strategies provide different gradients to recapitulate the physiological systems on the cellular, architectural, and biochemical levels to enhance differentiation and mineralisation in osteochondral tissue engineering.

### 4.2 Engineering organoids

Organoids are multicellular 3D miniaturised constructs of tissues or organs derived from stem and progenitor cells that recapitulate key functional aspects of its corresponding organ [284]. They are formed through the differentiation and self-organisation of pluripotent stem cells or progenitor cells, which can contain supporting stromal elements, and recapitulate the biochemical and biophysical cues in the native complex ‘niche’ for their proper function [285]. Compared to conventional 2D cultures or spheroids, organoids allow for more accurate investigations and reliable outcomes due to structural and functional resemblance to the corresponding organ. This offers a valuable tool to investigate the underlying mechanisms of human development which can be applied for personalised medicine, drug screening and cell therapy [286].

Organoid model systems have been successfully constructed from a range of organs [286]. Attempts have also been made to create trabecular bone-like organoids [287,288]. Organoids are commonly derived from pluripotent stem cells (PSCs), such as embryonic stem cells (ESCs) or iPSCs [284]. Matrigel, which is the reconstituted basement membrane extract from Englebreth-Holm-Swarm murine sarcoma cells [289], is commonly used as universal matrix for the generation of organoids. However, its undefined nature and heterogeneous mechanical properties interfere with the identification of key signals involved in organoid function [286].

Engineered matrices can be utilised to replace conventional organoid culture matrices, e.g. Matrigel, and enable control over the biophysical and biochemical cues presented. Biomaterials are chemically defined and can be engineered to provide better tuneability and low batch-to-batch variability. Therefore, a range of hydrogel-based systems are being explored for organoid cultures, including decellularised tissues [290], natural polymers (such as collagen [291] and alginate [292]), and synthetic polymers, including polyethylene glycol (PEG)-based hydrogels [293]. Recent advances in engineering organoid systems have also included poly(lactic-co-glycolic acid) (PLGA) microfilaments as ‘floating scaffolds’ [294], employing biofabrication techniques in organoid assembly [295], and ‘organoid-on-chip’ platforms [296].

Organoids have been applied to the study of various signalling pathways, including Hh signalling. Orthotopic transplantation of patient-derived organoids generated from resected tumour tissues resulted in histological characteristics which are similar to that observed in the patient's tumour tissue with regards to high *GLI1* expression, confirming the key role of Hh signalling in gastric cancer [297]. The role of Shh in regulating the regeneration of gastric epithelium in response to injury has also been investigated using human derived-gastric organoid and macrophages co-culture [298]. The chemo-attractant properties of Shh were associated with increased *GLI* and *PTCH1* expression in the fractured epithelium as an outcome of Smo-dependent activation route of Hh via cross-talk with PI3K/AKT signalling pathway [298].

Organoids have also been employed for drug screening. The role of Hh signalling in Sorafenib resistance has been investigated using patients-derived CD44^+^ hepatocellular carcinoma organoids which showed increased *GLI1*, *SMO* and *PTCH1* expression upon Sorafenib treatment. Inhibiting canonical Hh signalling with GANT61 (Gli1 and Gli2 inhibitor) significantly reduced Hh target gene expression in parallel with decreased CD44 expression leading to lower cell viability and reduced malignant properties [299]. Further investigations revealed the indirect involvement of PTCH-dependent-GLI1-independent, non-canonical Hh signalling in increasing the survival rate of cancer stem cells via cross-talk with Wnt signalling in patient-derived colon cancer organoids [300]. Moreover, the role of primary cilia, but not of canonical Hh signalling, in testicular morphogenesis has been confirmed in cyclopamine-treated organoids [301].

### 4.3 Engineering 3D scaffolds

Scaffold-free culture systems may fail to recapitulate cell-matrix interactions and the resulting intercellular signalling pathways that produce morphogen gradients. Research on Hh signalling has been mostly based on experiments carried out using 2D cell cultures. However, such 2D cultures pose numerous limitations, including the lack of biomimetic matrix cues and 3D cell-cell interactions, as well as changes in cell polarity and morphology. Employing scaffold-based cultures, for example using hydrogel-based and polymer-based scaffolds, offers a more physiologically relevant cellular environment and allows the user to introduce a range of physical cues in the model system.

A range of biodegradable biomaterials are available for the fabrication of scaffolds used in tissue engineering and regenerative medicine applications [[302], [303], [304]]. Hydrogels are generally prepared by translating hydrophilic polymers solution into 3D structures via physical or chemical cross-linking of the hydrophilic polymer chains. They can either be ECM-derived substrates, e.g. decellularised ECM isolated from animal tissues, or materials of natural (e.g. alginate) or synthetic (e.g. PEG) origin [305]. On the other hand, other polymers such as poly-α-hydroxy acids (e.g. polyglycolic acid (PGA), poly lactic acid (PLA) and their copolymer PLGA can provide stiffer scaffolds and have been used for MSCs expansion with high osteogenic gene expression profile and improved subsequent differentiation potential *in vivo* compared to other polymers [156,306,307]. Blends of polymers and nanoparticles can also be utilised to display tunable physical properties [308]. Silk-based matrices have also been utilised to regulate osteogenic signalling pathways [309]. The physiochemical properties of these scaffolds can be tuned to provide vital cues needed to control the function of the encapsulated cells and to modulate signalling pathways of interest, such as Hh signalling.

Biomimetic approaches for developing biomaterials for bone tissue engineering show great promise. There is a growing emphasis on novel *in vitro* model designs utilising surface-engineered scaffolds to achieve biomimetic physical environments [310,311]. Conventional 2D cultures can be designed to include a localised population of Shh ligand-producing cells, for example, but the extent of the influence of the secreted ligand was shown to differ between 2D and 3D cultures [312]. The more physiologically-relevant 3D models can be used to investigate how secreted Shh ligand is transported through extracellular spaces whilst remaining available to bind transmembrane receptors and initiate signal transduction. The inclusion of micron-scale surface topographical features on 3D scaffolds represents one complementary way to regulate cell response, independent of material biochemical properties [155,313].

Tailored cell culture scaffolds mimicking native bone matrix stiffness can be employed to study cell-matrix interactions *in vitro*. The addition of signalling biomolecules, such as growth factors, can provide additional biochemical cues found in the natural ECM environment [314], but do not also mimic the complexity of the multiscale topographical architectures that can influence signalling pathways [315]. For spatiotemporally-controlled biochemical patterning in a hydrogel, SHH and ciliary neurotrophic factor proteins have been immobilised using orthogonal physical binding pairs in a spatial orientation similar to their presentation *in vivo* to produce bioactive 3D-patterned agarose-based hydrogels. Adult neural precursor cells migrated along these patterned immobilised–SHH–gradients [316]. Strategies such as layer-by-layer assembly [317], freeze casting [318], and 3D printing [319,320] have been used to fabricate biomimetic, tailored structures similar to native bone tissue.

Scaffolds incorporating dynamic matrix stiffness are critical to study ECM stiffness-induced signalling [321]. Biomaterials can be modified to fabricate scaffolds with tuneable stiffness, ranging from softer tissue-like elasticity, such as bone marrow, to hard tissues such as bone [322]. To assess the influence of matrix stiffness on *Gli1* expression, Yu *et al.* altered the stiffness of a gelatin/polyacrylamide-based hydrogel platform. Higher expression levels of *Gli1* were detected in the mouse embryonic fibroblasts seeded on the stiffer hydrogels, which corresponded to increases in cell spreading area [162]. Stimuli-responsive hydrogels, which allow real-time manipulation of structural and mechanical properties in response to environmental triggers, can be utilised to provide mechanically-dynamic 3D environments, which will help answer fundamental questions regarding mechanical regulation of Hh signalling. Such hydrogels can be designed to respond to various stimuli, such as temperature, photo-activation, magnetic and electric fields, pH, as well as numerous biological and chemical cues [323,324].

Various studies have investigated the use of 3D scaffolds to maximise Hh pathway activation to induce bone regeneration. Vesicles-loaded with SAG (a Smo agonist) immobilised on apatite-coated-PLGA scaffolds were shown to induce osteogenic differentiation of BM-MSCs *in vitro* and enhanced the healing of size-specific bone-defects *in vivo* by increasing the expression of *Gli1* [325]. Moreover, Shh-loaded chitosan microspheres encapsulated into fibrin scaffolds is another approach that has been used for modulating Hh signalling to restore stem cell proliferation and enhance regeneration in spinal cord injury [326].

## 5 Concluding remarks

An ideal experimental model system will replicate key cellular and molecular interactions that can be tailored to manipulate Hh signalling without the use of exogenous small molecules, making it more suitable for high-throughput screening of novel drug candidates. Understanding the role of mechanical cues in the regulation of the Hh signalling pathway *in vitro* will only be achieved by examining the expression profile of Hh core components in physiologically-relevant 3D cultures in the absence of confounding biochemical factors that are typically added to induce differentiation. The incorporation of tailored micro-materials in developmental bioengineering applications will allow the modulation of self-organisation from within a developing construct with high reproducibility and reliability, presenting exciting new avenues for modulating cell behaviour, for example within organoids. Outcomes can be used to provide guiding parameters for the design of physical features of microscale 3D *in vitro* models of diseases associated with Hh signalling dysfunction and development-inspired bioengineering.

## Credit author statement

**F Ghuloum**: Writing – original draft, Funding acquisition. **C Johnson**: Supervision, Writing – review & editing. **N Riobo-Del Galdo**: Supervision, Writing – review & editing. **M Amer**: Conceptualization, Supervision, Funding acquisition, Writing – review & editing.

## Declaration of competing interest

The authors declare that they have no known competing financial interests or personal relationships that could have appeared to influence the work reported in this paper.

## Data Availability

No data was used for the research described in the article.
